# Healthcare Resource Utilisation Associated with Herpes Zoster in a Prospective Cohort of Older Australian Adults

**DOI:** 10.1371/journal.pone.0160446

**Published:** 2016-08-02

**Authors:** Surendra Karki, Anthony T. Newall, C. Raina MacIntyre, Anita E. Heywood, Peter McIntyre, Emily Banks, Bette Liu

**Affiliations:** 1 School of Public Health and Community Medicine, University of New South Wales (UNSW), Sydney, Australia; 2 National Centre for Immunisation Research and Surveillance of Vaccine Preventable Diseases, The Children's Hospital Network, Westmead, NSW, Australia; 3 National Centre for Epidemiology and Population Health, Australian National University, Canberra, ACT, Australia; 4 The Sax Institute, Sydney, Australia; University of Cambridge, UNITED KINGDOM

## Abstract

**Background:**

Herpes zoster (HZ) is a common condition that increases in incidence with older age but vaccines are available to prevent the disease. However, there are limited data estimating the health system burden attributable to herpes zoster by age.

**Methods:**

In this study, we quantified excess healthcare resource usage associated with HZ during the acute/sub-acute period of disease (21days before to 90 days after onset) in 5952 cases and an equal number of controls matched on age, sex, and prior healthcare resource usage. Estimates were adjusted for potential confounders in multivariable regression models. Using population-based estimates of HZ incidence, we calculated the age-specific excess number of health service usage events attributable to HZ in the population.

**Results:**

Per HZ case, there was an average of 0.06 (95% CI 0.04–0.08) excess hospitalisations, 1.61 (95% CI 1.51–1.69) excess general practitioner visits, 1.96 (95% CI 1.86–2.15) excess prescriptions filled and 0.11 (95% CI 0.09–0.13) excess emergency department visits. The average number of healthcare resource use events, and the estimated excess per 100,000 population increased with increasing age but were similar for men and women, except for higher rates of hospitalisation in men. The excess annual HZ associated burden of hospitalisations was highest in adults ≥80 years (N = 2244, 95%CI 1719–2767); GP visits was highest in those 60–69 years (N = 50567, 95%CI 39958–61105), prescriptions and ED visits were highest in 70–79 years (N = 50524, 95%CI 40634–60471 and N = 2891, 95%CI 2319–3449 respectively).

**Conclusions:**

This study provides important data to establish the healthcare utilisation associated with HZ against which detailed cost-effectiveness analyses of HZ immunisation in older adults can be conducted.

## Introduction

Herpes zoster (HZ), commonly known as “shingles”, is a viral disease that occurs as a result of reactivation of latent varicella zoster virus (VZV) established in the spinal dorsal root or cranial ganglia after primary infection [1]. Acutely, it is usually characterised by unilateral, painful, vesicular, dermatomal eruptions along a single spinal or cranial sensory ganglia [2]. In addition to acute manifestations, around 10–20% of patients in the unvaccinated population experience chronic pain, also known as post-herpetic neuralgia (PHN), that can seriously affect quality of life [3–5]. PHN is variably defined as pain persisting for 1 month [6], 6 weeks [7], 2 months [8], 3 months [3, 9] and 6 months after the HZ rash is healed [10]. However, from the perspective of measuring healthcare resource utilisation, the first three months after the onset of HZ has been considered the acute/sub-acute phase and the chronic phase is defined as persistence of sensory symptoms more than 3 months after the onset of HZ vesicular eruptions [3, 9, 11–13].

The incidence of HZ and PHN markedly increases with age, particularly after 50 years [14, 15]. In people aged over 50 years, annually approximately 60,000 new cases of HZ occur in Australia, an incidence of 10–12 per 1000 population [5, 16]. Both HZ and PHN can be prevented by immunisation. A live attenuated HZ vaccine is licensed for use in adults 50 years and older in Australia and Europe and in those aged 60 years and above in the US [17, 18]. In Australia, commencing in November 2016, this vaccine will be provided free of charge to people aged 70 years under the National Immunisation Program (NIP), with a time limited catch up program for people aged 71–79 years [19].

Previous studies from US have reported on HZ associated healthcare resource usage in adults however these have been based on insurance claims databases [11, 12, 20]. The comprehensiveness of these data depend on the type of population covered under the insurance scheme, the accuracy of coding, and decision algorithms to attribute the ‘possibly related non-specific codes’ to illness due to HZ. Studies from Australia have described resource usage but only for cases only data and without detail on patient characteristics [5, 21]. Studies that estimate excess healthcare resource utilisation without an appropriate comparator group may be confounded by levels of prior comorbidity among HZ cases. In this study, we use a large-scale population-based cohort linked to records of hospitalisations, general practitioner (GP) visits, prescriptions, and emergency department (ED) visits to estimate the excess healthcare resource utilisation during the acute/sub-acute period in HZ cases compared to controls matched and adjusted on a range of factors including prior healthcare resource usage.

## Methods

### Setting, design and participants

We compared healthcare resource utilisation between cases of HZ and controls with no evidence of recent HZ, identified from a cohort of 267,000 participants aged ≥45 years and prospectively followed up in the state of New South Wales (NSW), Australia (the Sax Institute’s 45 and Up Study). A detailed description of the 45 and Up study cohort is published elsewhere [22]. Briefly, adults aged ≥45 years and residing in NSW were randomly selected from the Australian Medicare enrolment database, which covers virtually all of the population, and invited to participate in the study. The cohort includes about 10% of all adults living in the state within the targeted age range [22]. All participants completed a questionnaire at recruitment that included information on socio-demographic, behavioural and health related information and consented to long-term follow up and linkage of their information to a wide-range of other healthcare-related administrative databases [22].

The cohort was followed up for hospitalisations, general practitioner (GP) visits, prescriptions filled, emergency department (ED) visits, and deaths via record linkage to the NSW Admitted Patient Data Collection (APDC), Medicare Benefits Schedule (MBS), Pharmaceutical Benefits Scheme (PBS), NSW Emergency Department Data Collection (EDDC) and the NSW Registry of Births, Deaths and Marriages (RBDM), respectively. The NSW Centre for Health Record Linkage (CHeReL) performed the record linkage to the APDC, EDDC and RBDM using probabilistic matching [23]. The Sax Institute provided the Department of Human Services (DHS) with unique identifier data of the 45 and Up study and DHS provided MBS and PBS data according to the unique identifier. The PBS, and MBS data were later linked to the 45 and Up study data by the Sax Institute. All linkages were conducted independently of the study investigators. The APDC database records every hospitalization in NSW including information on the date of admission and discharge, the primary diagnosis related to hospitalization, and up to 49 secondary diagnoses affecting treatment or length of stay coded according to the International Classification of Disease 10^th^ revision, Australian Modification (ICD-10-AM) [24]. APDC data was used to ascertain and count the number of hospitalisations. The MBS data records claims for subsidised medical care under the Medical Benefits Schedule (MBS), part of Australia’s universal healthcare insurance system (Medicare) which includes GP attendances [25, 26]. MBS data was used to ascertain and count the number of GP visits and total number of MBS claims. Similarly, PBS data record claims on subsidised pharmaceutical products dispensed under the Pharmaceutical Benefits Scheme [27, 28]. PBS data was used to measure the total number of prescriptions filled as well as identify specific prescriptions such as the number of antidepressant or anticonvulsants. The EDDC records emergency department (ED) visits by NSW residents in large public hospitals, covering a majority of the population [29]. EDDC data was used to measure the number of ED visits made by participants during the study period. The RBDM records the date of all births, deaths and marriages in NSW residents [30]. Complete records for study participants from the APDC, MBS, PBS, EDDC and RBDM database were available up to 31 December 2011.

### Study definitions

A detailed methodology on HZ case ascertainment, along with factors associated with HZ is described elsewhere [16]. In brief, participants who had records of either specific antiviral medication for treatment of HZ in the PBS database (1052J, 8002E, 8064 K, 8897G) or a record of hospitalisation coded as “B02-Zoster” (ICD-10 codes) in either the principal or secondary diagnosis field in the APDC database following recruitment were defined as “HZ cases”. To ensure the inclusion of only incident cases, those participants who had a record of a HZ diagnosis prior to their recruitment date were excluded. In addition, to allow for sufficient follow-up of health service use after the onset of HZ, only incident cases diagnosed before 31 December 2010 were included in the analyses.

Definitive attribution of all healthcare resource usage due to HZ is usually impractical due to insufficient clinical detail in large electronic databases such as those linked in this study. Hence, we compared the overall resource usage within each domain of interest (number of hospitalisations, GP visits (defined as MBS item numbers 3, 4, 20, 23, 24, 35, 36, 37, 43, 44, 47, 51), prescriptions filled, and ED visits) to estimate the excess healthcare resource usage. Similar to other studies, we defined the “acute/sub-acute phase” as a period of 21 days prior to and 90 days following the diagnosis of HZ [12]. We also created two time periods of equivalent length (112 days) before and after the “acute/sub-acute” phase (altogether 5 periods, P1 (-245 to -134 days), P2 (-133 to -22 days), P3-acute/sub-acute period (-21 to +90 days, day 0 represents the HZ diagnosis day), P4 (+91 to +202 days), P5 (+203 to +314 days), hereafter called the analysis time-frame) and compared the resource utilisation in corresponding periods between cases and controls.

For the primary analyses, we selected matched controls equal in number to the HZ cases. Controls were selected from the cohort and matched to cases on age (single year), sex, and prior health resource usage. Prior resource usage was estimated by taking the average number of GP visits, hospitalisations, prescriptions filled and ED visits in the one year period (day -610 to -246) prior to the analysis time-frame. All participants in the cohort who were not cases were randomly assigned an index date within the range of first and last date of zoster diagnosis observed among the cases. Propensity scores were then calculated using the matching variables, and controls were selected based on those with the closest match on propensity score to cases. Both cases and controls had equivalent periods of resource usage data available.

### Statistical analyses

Average use of healthcare resources was plotted for cases and controls up to 360 days before and after the onset of HZ. Poisson regression was used to model the relative increase in the number of hospitalisations, GP visits, prescriptions filled and ED visits compared to the equivalent time-period in the matched controls. The regression analyses were adjusted for prior utilisation of resources to adjust for any residual difference after matching, and all other variables that were significantly associated with a HZ diagnosis as described in a previous publication [16]. These variables included: age; sex; residency (major city, inner regional, outer regional/remote), partner/marital status, smoking, attended preventative health screening, supplements taken 4 weeks prior to recruitment, prior immune suppression, prior cancer, physical limitation (none, minor, moderate, severe), heart disease and asthma.

The average number of events per HZ case and control, and adjusted relative and absolute increase in number of events in HZ cases versus controls was estimated over the acute /sub-acute phase. We then used data on HZ incidence from a nationally representative sample of Australian adults attending general practices [21] (Appendix A in S1 File) to calculate the excess healthcare resource utilisation per 100,000 population and applied this to 2014 Australian projected population data [31] to derive estimates of total annual healthcare resource use attributable to HZ.

In addition to analyses of hospitalisations, GP visits, PBS items and ED visits, we also performed analyses examining excess total MBS claims (which include GP visits, specialist visits, and other services funded under the MBS) and specific PBS items that may be associated with a more severe illness (analgesics and antidepressant drugs: anatomical therapeutic chemical (ATC) classification system codes N02A [opioids], N02BE [anilides] and N06A [antidepressants]) [32].

### Sensitivity analyses

As our definition of HZ cases included those people who were hospitalised with a diagnosis of HZ but may not have a record of being dispensed antivirals for HZ, we conducted a sensitivity analysis excluding those cases that were identified based only on hospital records. We also compared resource utilisation using self-matched analyses (cases only). In these analyses, the relative increase in resource use during the acute/sub-acute period was compared to a prior period of 112 days. These case-only analyses were adjusted for the underlying increase in resource use that occurs with increasing age (see Appendix B in S1 File for details).

All analyses were conducted in STATA 12 software (StataCorp, College Station, Texas, USA). All participants provided written informed consent. The conduct of the 45 and Up Study was approved by the University of New South Wales Human Research Ethics Committee (HREC). This specific study was approved by the NSW Population Health Research Ethics Committee, and the University of New South Wales Human Research Ethics Committee (2010/12/292).

## Results

**Fig 1:**
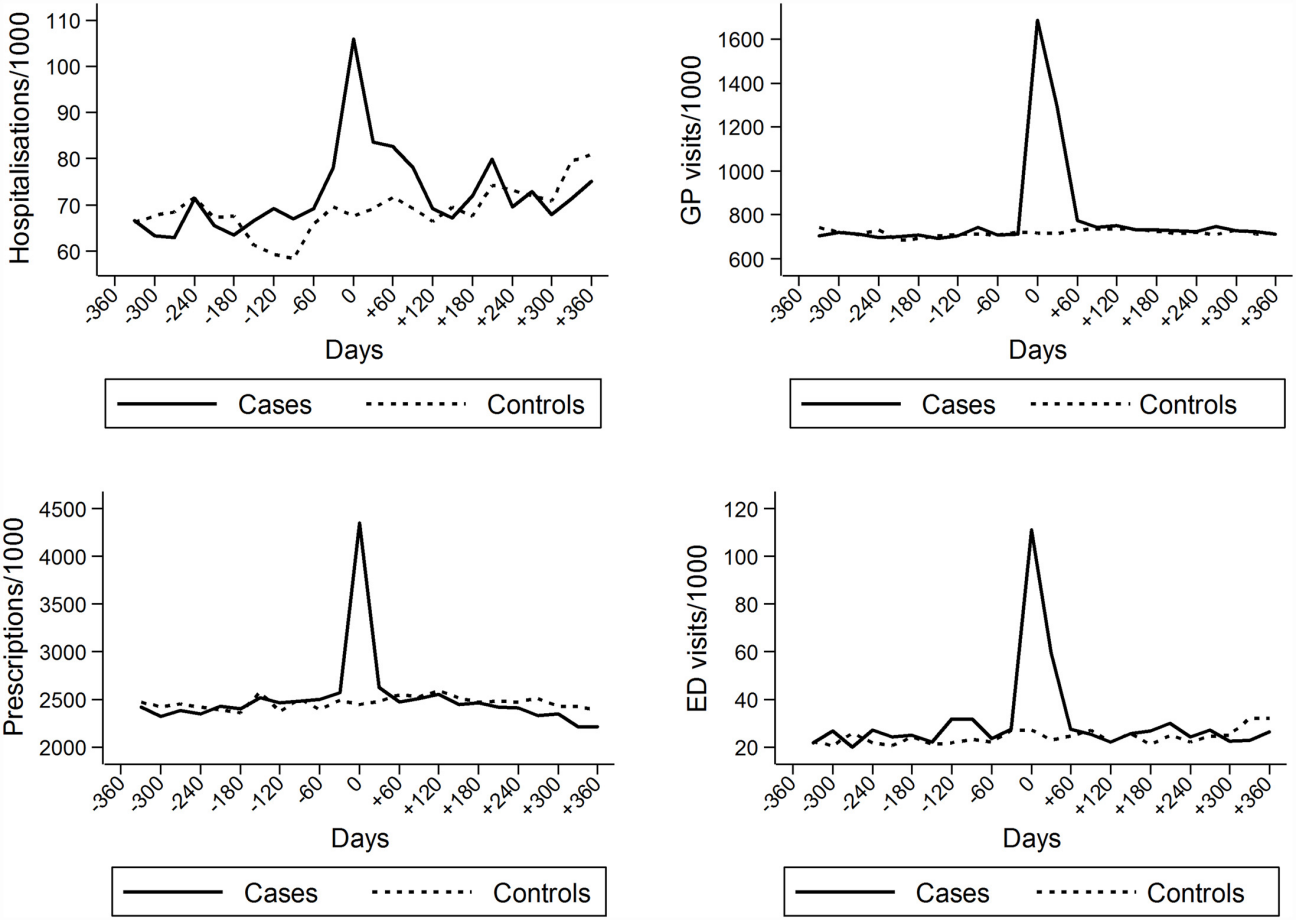
Healthcare resource usage in herpes zoster cases and controls per 1000 population in relation to time at diagnosis or index day (day = 0). Note: ED, emergency department, GP, general practitioner.

**Fig 2:**
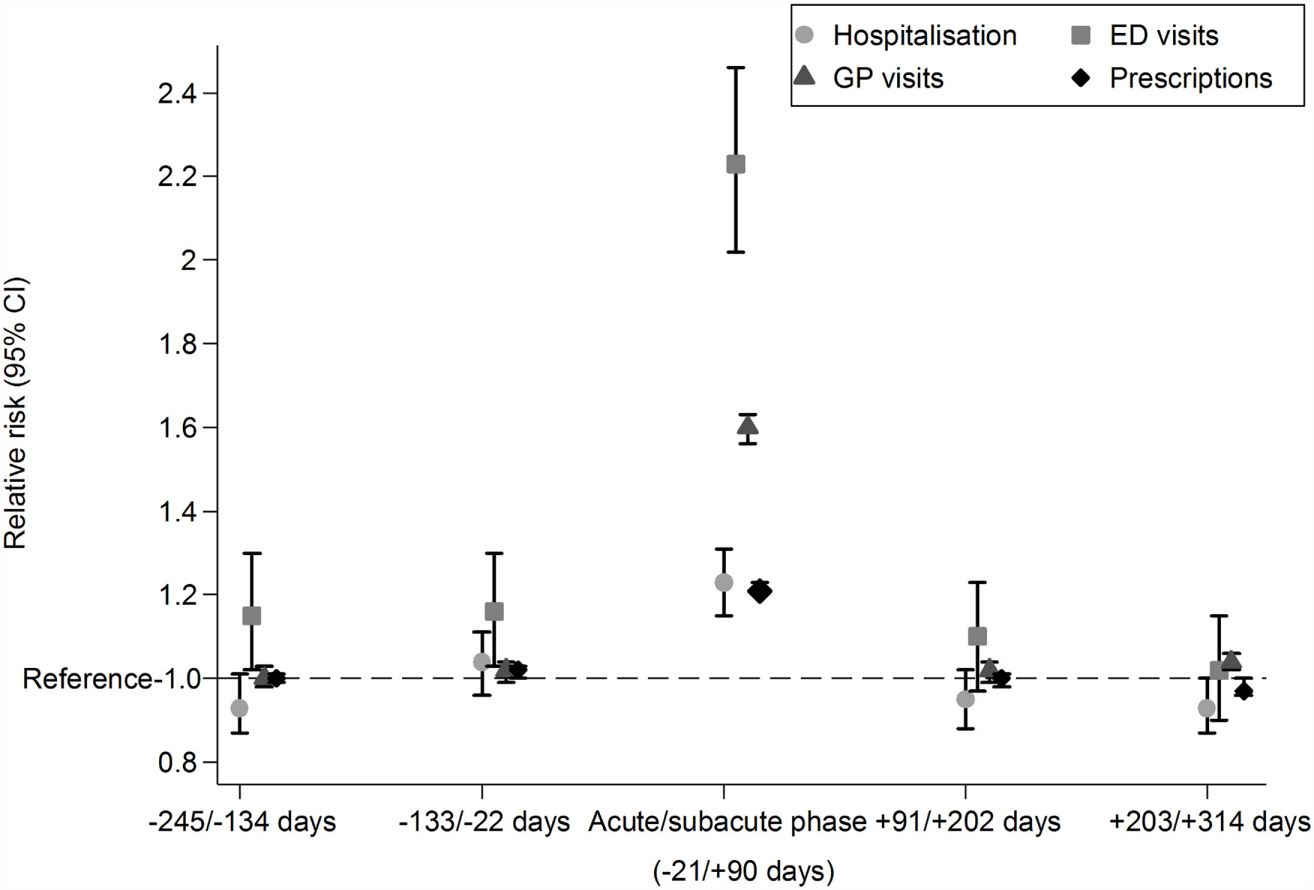
Relative increase in healthcare resource usage in herpes zoster cases compared to controls in acute/sub-acute phase and equivalent time periods before and after. Note: CI, confidence interval, ED, emergency department, GP, general practitioner.

A total of 5,952 cases and an equal number of controls matched on prior healthcare resource usage, age and sex were included in the analyses. Characteristics of cases and controls included in the analyses are shown in Table 1. Controls were slightly older than cases in each age category. Prior hospitalisations, GP visits, prescriptions and ED visits were similar. Fig 1 shows the utilisation of healthcare resources per 1000 cases and controls within the analysis time-frame. The bulk of the excess resource utilisation occurred in acute/sub-acute period of HZ. Fig 2 shows the relative increase in resource utilisation in HZ cases compared to controls in equivalent time periods. Significant increases comparing cases to controls were only observed during the acute/sub-acute period except for ED visits where a significant increase was also observed in period immediately prior to the acute/sub-acute phase.

**Table 1:** Characteristics of cases with herpes zoster and matched controls.

Characteristics	Cases n = 5952	Controls n = 5952
**Age in years-N (mean, SD)**		
45–59	1831 (53.0, 3.6)	2011 (53.5, 3.9)
60–69	1814 (63.4, 3.0)	1785 (64.4, 3.2)
70–79	1432 (73.1, 3.0)	1400 (74.1, 3.3)
80+	875 (82.8, 3.6)	756 (83.3, 3.5)
**Sex- N (%)**		
Male	2366 (39.8)	2366 (39.8)
Female	3586 (60.2)	3586 (60.2)
**Residency- N (%)**		
Major city	2871(48.2)	2775 (46.6)
Inner regional	2003 (33.6)	2068 (34.7)
Outer regional	980 (16.5)	967 (16.2)
Remote	95 (1.6)	135 (2.3)
**Partner/Married- N (%)**		
Yes	4429 (74.4)	4310 (72.4)
No	1523 (25.6)	1642 (27.6)
**Smoking- N (%)**		
Never	3444 (57.9)	3430 (57.6)
Past	2179 (36.6)	2076 (34.9)
Current	302 (5.1)	4.11 (6.9)
**Attended preventative health screening**^a^**- N(%)**		
Yes	5343 (89.8)	5355 (90.0)
No	609 (10.2)	597 (10.0)
**Supplements in 4 weeks prior to recruitment- N(%)**		
Yes	5278 (88.7)	5390 (90.6)
No	674 (11.3)	562 (9.4)
**Prior immunosuppression**^b^**- N(%)**		
Yes	106 (1.8)	82 (1.4)
No	5846 (98.2)	5870 (98.6)
**Prior cancer- N(%)**		
Yes	483 (8.1)	348 (5.8)
No	5469 (91.9)	5604 (94.2)
**Physical limitation- N(%)**		
None	1397 (23.5)	1384 (23.3)
Minor	865 (14.5)	785 (13.2)
Moderate	1836 (30.9)	1908 (32.1)
Severe	1210 (20.3)	1214 (20.4)
**Heart disease- N(%)**		
Yes	961 (16.2)	1051 (17.7)
No	4991 (83.8)	4901 (82.3)
**Asthma- N(%)**		
Yes	910 (15.2)	916 (15.4)
No	5042 (84.7)	5036 (84.6)
**Prior health care utilisation -N (mean, SD)**^c^		
Hospitalisation	5952 (0.8, 4.2)	5952 (0.8, 5.5)
GP visits	5952 (8.4, 7.4)	5952 (8.2, 7.1)
Prescriptions filled	5952 (27.6, 32.4)	5952 (27.4, 31.7)
ED visits	5952 (0.3, 0.9)	5952 (0.3, 0.8)

Columns do not always add to totals due to missing data

^a^ Any of prostate specific antigen testing, mammography, bowel cancer screening

^b^ Prior immunosuppression was defined if individuals linked to a HIV registration, a PBS record of an immunosuppressive medication, or a hospitalization in the year prior to study entry for any one of a list of potentially immunosuppressive conditions including transplants or conditions that may result in administration of high-dose steroids [16].

^c^ Per unit of time—see methods

Abbreviations: SD, standard deviation; GP, general practitioner; ED, emergency department.

Table 2 shows results from the regression analyses adjusted for age and other factors outlined in the methods. During the acute/sub-acute period compared to controls, per HZ case there was an average excess of 0.06 (95% CI 0.04–0.08) hospitalisations, 1.61 (95% CI 1.51–1.69) GP visits, 1.96 (95% CI 1.86–2.15) prescriptions filled and 0.11 (95% CI 0.09–0.13) ED visits. The average number of health service use events, and the estimated excess increased with increasing age. For example the average excess in hospitalisations ranged from 0.03 (95% CI 0.01–0.06) in 45–59 year olds to 0.16 (95% CI 0.06–0.27) in those ≥80 years. Once HZ incidence was taken into account, the estimated excess health service use associated with HZ per 100,000 population also increased with age (see Table 2). Comparing those aged ≥80 years to 45–59 year olds, excess hospitalisations were more than 10-fold greater, excess GP visits more than three-fold greater, excess prescriptions six-fold greater, and excess ED visits four-fold greater. When applied to Australian projected population data we found the annual number of hospitalisations attributable to HZ were highest in those ≥80 years, GP visits were highest in 60–69 year olds, and prescriptions and ED visits were highest in 70–79 year olds (see Table 2, last column). The excess resource utilisation for GP visits, prescriptions and ED visits were similar for men and women; however, the hospitalisation rate was higher in men (adjusted RR 1.42 (95%CI 1.29–1.56) versus 1.09 (1.00–1.20); see Appendix C in S1 File).

**Table 2:** Estimated excess healthcare usage (excess hospitalisations, general practitioner visits, prescriptions, and emergency department visits) due to herpes zoster in period 21 days prior and until 90 days after onset.

Variable	Mean events/ case (SE)	Mean events/control (SE)	Adjusted relative risk of event (95% CI)	Adjusted excess events/case^a^ (95% CI)	Adjusted excess events/100,000^b^ (95% CI)	Excess events in Australian population^c^ (95%CI)
**Hospitalisations**						
Age groups						
45–59 years	0.17 (0.027)	0.12 (0.010)	1.24 (1.04–1.47)	0.03 (0.01–0.06)	19 (15–22)	860 (679–996)
60–69 years	0.27 (0.023)	0.20 (0.032)	1.35 (1.17–1.55)	0.07 (0.03–0.11)	60 (47–73)	1430 (1120–1740)
70–79 years	0.44 (0.060)	0.39 (0.068)	1.24 (1.10–1.40)	0.09 (0.04–0.16)	136 (109–162)	1947 (1560–2319)
≥80 years	0.62 (0.096)	0.61 (0.137)	1.26 (1.10–1.44)	0.16 (0.06–0.27)	248 (190–306)	2244 (1719–2767)
**General practitioner visits**						
Age groups						
45–59 years	3.29 (0.063)	1.90 (0.045)	1.74 (1.67–1.80)	1.41 (1.27–1.52)	917 (740–1094)	41529 (33513–49544)
60–69 years	4.03 (0.068)	2.48 (0.061)	1.67 (1.60–1.73)	1.66 (1.49–1.81)	2121 (1676–2563)	50567 (39958–61105)
70–79 years	4.94 (0.083)	3.35 (0.075)	1.52 (1.47–1.58)	1.74 (1.57–1.94)	2526 (2031–3022)	36154 (29069–43253)
≥80 years	5.94 (0.135)	4.02 (0.120)	1.49 (1.43–1.56)	1.97 (1.73–2.25)	3075 (2354–3798)	27824 (21300–34366)
**Prescriptions**						
Age groups						
45–59 years	4.57 (0.185)	3.55 (0.169)	1.33 (1.29–1.37)	1.17 (1.03–1.31)	764 (616–911)	34600 (27897–41259)
60–69 years	10.67 (0.275)	9.11 (0.275)	1.25 (1.22–1.28)	2.28 (2.00–2.55)	1954 (1544–2362)	46585 (36811–56312)
70–79 years	16.13 (0.355)	14.32 (0.354)	1.17 (1.15–1.20)	2.43 (2.15–2.86)	3530 (2839–4224)	50524 (40634–60471)
≥80 years	18.73 (0.467)	16.09 (0.500)	1.20 (1.17–1.23)	3.22 (2.73–3.70)	5023 (3846–6204)	45450 (34800–56136)
**Emergency department visits**						
Age groups						
45–59 years	0.15 (0.014)	0.07 (0.007)	2.11 (1.72–2.59)	0.08 (0.05–0.11)	51 (41–60)	2310 (1857–2717)
60–69 years	0.20 (0.014)	0.08 (0.009)	2.57 (2.10–3.14)	0.13 (0.09–0.17)	108 (85–130)	2575 (2026–3099)
70–79 years	0.24 (0.017)	0.10 (0.012)	2.39 (1.96–2.92)	0.14 (0.10–0.19)	202 (162–241)	2891 (2319–3449)
≥80 years	0.33 (0.025)	0.18 (0.019)	1.83 (1.49–2.25)	0.15 (0.09–0.22)	233 (179–288)	2108 (1620–2606)

Note: Relative risks are given for cases versus controls and adjusted for prior number of hospitalisations, GP visits, prescriptions, and ED visits; age; sex; residency, partner/married, smoking, attended preventative health screening, taken supplements 4 weeks prior to recruitment, prior immune suppression, prior cancer, physical limitation, heart disease and asthma (see methods).

^a,b, c^ see methods for estimation.

In sensitivity analyses including only cases of HZ who were diagnosed on the basis of HZ specific antiviral medications (N = 5847), a significant increase in hospitalisations per HZ case remained although the increases were smaller (Appendix D in S1 File).

In separate analyses, which accounted for all claims listed in the MBS database (including GP and other provider visits), there were an average of 3.47 (95% CI 3.36–3.70) excess MBS items recorded during the acute/sub-acute period per case of HZ. Excess MBS items also increased with increasing age (Appendix D in S1 File).

There were an average of 0.47 (95% CI 0.43–0.51) excess prescriptions filled for analgesic drugs and 0.11 (95% CI 0.08–0.14) antidepressant drugs recorded during the acute/sub-acute period per HZ case (Appendix D in S1 File). A slight excess in prescriptions of analgesics, anticonvulsants and antidepressants were also evident after the acute/sub-acute period of HZ (Appendix E in S1 File).

Finally in the case-only analyses for hospitalisations, GP visits, prescriptions and ED visits the results were consistent with the estimates obtained with the case-control method (Appendix F in S1 File).

## Discussion

In this study, we found that compared to matched controls, at a population-level, HZ results in substantial increases in hospitalisations, GP visits, prescriptions of medication and ED visits in the acute/sub-acute period of HZ. Taking disease incidence into account, rates of excess healthcare resource usage increased considerably with increasing age with people 80 years and older having the highest excess utilisation rate per 100,000. When estimating the total burden of resource usage in healthcare system, excess healthcare usage was substantial in both age groups currently eligible for free vaccine in Australia (70–79 years) as well as in age groups not currently eligible, for example those ≥80 years, and those 60–69 years.

Our study also showed that most of the excess healthcare usage occurred in the acute/sub-acute period (up to 3 months after a diagnosis) with only ED visits significantly greater in HZ cases compared to controls in the time period before the acute/sub-acute period (Fig 2). In additional analyses, only the prescriptions of analgesics, anticonvulsants and antidepressants remained increased for a longer term (Appendix E in S1 File). Other studies have shown that a substantial increase in utilisation of healthcare resources occurs among patients with PHN or non-pain complications after the first 3 months of disease onset [20, 33]. One of the reasons that the long-term increase in resource use was not evident in our overall analyses may be due to the fact that about 61% of the cases were under 70 years of age and the incidence of PHN is lower in these age groups. However in an exploratory analysis focussed on only those aged over 70 years at diagnosis (data not shown), we did not find substantial increases in resource use after the first 3 months. It could also be because we used non-specific measures of health service use (such as total GP visits) rather than items known to be definitely attributable to HZ.

Various approaches have been used to estimate the excess resource utilisation associated with HZ. White [11] and Dworkin [20] used regression based approaches considering all services provided to patients from US insurance claims data, whereas Insinga [12] used a regression based approach considering only selected conditions/services that are more likely to be due to HZ, however they cautioned that considering only the selected conditions thought to be due to HZ may underestimate the true resource utilisation. Yawn [33] used medical record review of case only data to attribute resource utilisation to HZ within 3 weeks before and up to 1 year after the onset of HZ. Previous studies conducted in Australia have used data collected in HZ cases only [5, 21].

Although our results derived from regression analyses of non-specific resource use comparing HZ cases to controls, they were largely consistent with findings from other studies conducted in Australia [5, 21]. For example a recent report on estimates of age specific hospitalisation rates attributable to HZ showed that excess hospitalisations in those with HZ are almost 16 times greater in those ≥80 years compared to 50–59 year olds [21]. Compared to our findings another Australian study estimated slightly higher numbers of general practitioner visits to manage a case of HZ [5] but this may be due to the fact that the study followed up cases until the complete management of HZ, whereas our study was limited to the acute/sub-acute period. Another study using claims data in the US reported slightly lower estimates for inpatient admissions, ED visits, and prescriptions compared to our study, which may be due to the fact that they selected only services which they considered more likely due to HZ [12]. Consistent with Insinga [12] the excess resource utilisation in our study was similar for males and females.

Compared to other Australian data, our study has some distinctive features. As HZ is likely to occur more often in the elderly and in those with pre-existing illnesses [16], our use of controls who were matched to cases on prior health service usage allowed pre-existing co-morbidities to be accounted for in estimating the excess health service use that could be attributed to zoster. In addition, our cohort has information on other variables and comorbidities that were adjusted for in regression analyses. However, several limitations should be noted in interpreting the finding of this study. The cases were ascertained using a record of being prescribed a HZ specific antiviral drug and hospitalisations coded as HZ, however individuals who visited the healthcare service too late to be prescribed antivirals may have been misclassified as controls. Also, we attributed the healthcare resource utilisation using regression analyses considering all resource utilisation listed in respective databases rather than through direct attribution using chart review. We cannot excluded the possibility that cases and controls differed in terms of some unmeasured confounders; however, case-only analyses showed similar results to those based on case-control comparisons, making it unlikely that this issue had a substantive impact on the results. Finally, for most participants the PBS data include only records of prescriptions above a co-payment amount [34] and not all prescriptions are filled. While this would be consistent between controls and cases, and therefore the relative excess estimates would be robust, but the absolute numbers of prescriptions are likely to be underestimates of overall prescription medicines used.

Given the methods applied, calculating the costs of excess healthcare use associated with HZ was beyond the scope of this study. However, as examples for illustration, the fee for a standard GP consultation in Australia is $37.05 [25] and the cost associated with the most frequent Australian refined diagnosis-related groups (AR-DRG, B72B) given to those with a principal hospital diagnosis of herpes zoster (ICD 10 code, B02.*) is $7260.00 [35]. Applying these unit costs to our estimates of excess hospitalisations and GP visits attributable to HZ in adults aged 45 years and above translates to an annual excess of $47 million in hospitalisations and $5.8 million in GP consultations in Australia.

In summary, our results demonstrate that HZ accounts for substantial excess resource use, including in age groups not covered by the National Immunisation Program. Given that new vaccines for HZ are now on the horizon [36], the data presented here can be useful for health services policy and planning, including cost-effectiveness analyses of HZ immunisation in older adults.

## Supporting Information

S1 File Appendices. (DOCX)
