# Supplementary material for: Healthcare Resource Utilisation Associated with Herpes Zoster in a Prospective Cohort of Older Australian Adults
Source: PLoS One. 2016 Aug 2;11(8):e0160446. doi: 10.1371/journal.pone.0160446 (PMC4970804; doi:10.1371/journal.pone.0160446)
Supplement: S1 File — (DOCX) [file pone.0160446.s001.docx]

**Appendices**

Appendix A: Incidence of herpes zoster in Australian population derived from Bettering the evaluation and care of health (BEACH) data.

| Age groups | Incidence per 1000 persons per year (95% CI) |
| --- | --- |
| Overall | 9.67 (8.66-10.68) |
| 45-59 years | 6.52 (5.26-7.78) |
| 60-69 years | 8.58 (6.78-10.37) |
| 70-79 years | 14.50 (11.66-17.35) |
| ≥80 years | 15.61 (11.95-19.28) |

Appendix B

For the self-matched analyses, similar to the main analyses, we created 2 periods each of 112 days before and after the acute/sub-acute period of HZ (a total of 5 periods (P1, P2, P3-acute/sub-acute period, P4, P5), spanning from -245 to +314 day from the date of diagnosis of HZ, hereafter called the analysis time-frame). We then created another 5 periods, each spanning 112 days, before the analysis time-frame (day -806 to -246 before the diagnosis of HZ). As expected, we observed a small but significant increase in resource usage over day -806 to -246 for hospitalisations, GP visits, and prescriptions but not for ED visits. In order to take into account the underlying effect of this increasing trend on the relative risk estimates during the acute/sub-acute period, we quantified the trend using Poisson regression for the period -806 to -246 days prior to onset of HZ as a percentage increase in health service use per 112 days. Then, the number of hospitalisation, GP visits, and prescriptions in each of the 5 periods within the analysis time-frame were adjusted for this. To account for repeated measures on the same individuals, clustered robust standard errors were calculated. Although relative risks and excess health service use were calculated for P2, P3-acute/sub-acute phase, P4, and P5 assuming P1 as reference period, only the results of acute/sub-acute phase are shown in Appendix VI as estimates for all other phases were similar to each other.

Appendix C: Estimated excess healthcare resource usage due to herpes zoster in the period 21 days prior and until 90 days after onset in men and women

| Variable | Cases (n) | Controls (n) | Average number of event/ cases (SE) | Average number of event/control (SE) | Relative risk (95%CI) |
| --- | --- | --- | --- | --- | --- |
| **Hospitalisation** |  |  |  |  |  |
| Men | 2366 | 2366 | 0.45 (0.050) | 0.34 (0.050) | 1.42 (1.29-1.56) |
| Women | 3586 | 3586 | 0.25 (0.018) | 0.23 (0.027) | 1.09 (1.00-1.20) |
| **GP visits** |  |  |  |  |  |
| Men | 2366 | 2366 | 4.34 (0.069) | 2.71 (0.058) | 1.60 (1.55-1.65) |
| Women | 3586 | 9580 | 4.28 (0.052) | 2.67 (0.043) | 1.60 (1.56-1.64) |
| **Prescriptions** |  |  |  |  |  |
| Men | 2366 | 2366 | 11.23 (0.257) | 9.10 (0.241) | 1.25 (1.22-1.27) |
| Women | 3586 | 3586 | 11.33 (0.213) | 9.51 (0.207) | 1.20 (1.18-1.21) |
| **ED visits** |  |  |  |  |  |
| Men | 2366 | 2366 | 0.23 (0.013) | 0.11 (0.009) | 2.14 (1.84-2.49) |
| Women | 3586 | 3586 | 0.20 (0.010) | 0.09 (0.006) | 2.30 (2.02-2.63) |

Abbreviations: SE, standard errors; CI, confidence interval; ED, emergency department, GP, general practitioner

Appendix D: Sensitivity analyses showing excess health service utilisation in herpes zoster (HZ) cases during acute/sub-acute disease phase (21 days prior and 90 days after the index date)

| Variable | Average number of events/case (SE) | Average number of events/control (SE) | Relative risk (95%CI) | Excess events per HZ case (95% CI) | Excess events/100000 persons (95% CI) |
| --- | --- | --- | --- | --- | --- |
| **Hospitalisation^a^** |  |  |  |  |  |
| Age groups |  |  |  |  |  |
| 45-59 years | 0.15 (0.015) | 0.12 (0.009) | 1.12 (0.94-1.34) | 0.01 (-0.01-0.04) | - |
| 60-69 years | 0.24 (0.017) | 0.21 (0.017) | 1.23 (1.06-1.42) | 0.05 (0.01-0.09) | 41 (33-50) |
| 70-79 years | 0.39 (0.030) | 0.39 (0.033) | 1.15 (1.01-1.30) | 0.06 (0.004-0.12) | 85 (68-101) |
| 80+ years | 0.53 (0.050) | 0.61 (0.067) | 1.20 (1.04-1.39) | 0.12 (0.03-0.24) | 190 (146-235) |
| **Total MBS items^b^** |  |  |  |  |  |
| Age groups |  |  |  |  |  |
| 45-59 years | 10.25 (0.308) | 7.76 (0.065) | 1.31 (1.28-1.34) | 2.40 (2.17-2.64) | 1568 (1265-1872) |
| 60-69 years | 13.68 (0.358) | 9.88 (0.074) | 1.39 (1.36-1.41) | 3.85 (3.57-4.05) | 3306 (2612-3996) |
| 70-79 years | 18.13 (0.571) | 13.95 (0.098) | 1.31 (1.28-1.33) | 4.32 (3.91-4.60) | 6271 (5042-7503) |
| 80+ years | 20.90 (0.775) | 16.60 (0.136) | 1.27 (1.24-1.29) | 4.48 (3.98-4.81) | 6996 (5356-8641) |
| **Analgesics** |  |  |  |  |  |
| Age groups |  |  |  |  |  |
| 45-59 years | 0.27 (0.031) | 0.16 (0.021) | 1.76 (1.51-2.04) | 0.12 (0.08-0.17) | 79 (64-95) |
| 60-69 years | 0.71 (0.043) | 0.34 (0.045) | 2.28 (2.06-2.52) | 0.43 (0.36-0.52) | 373 (295-451) |
| 70-79 years | 1.20 (0.066) | 0.35 (0.039) | 3.43 (3.09-3.80) | 0.85 (0.73-0.98) | 1233 (992-1476) |
| 80+ years | 1.32 (0.087) | 0.57 (0.079) | 2.42 (2.17-2.71) | 0.81 (0.67-0.97) | 1263 (967-1561) |
| **Antidepressants** |  |  |  |  |  |
| Age groups |  |  |  |  |  |
| 45-59 years | 0.32 (0.032) | 0.33 (0.030) | 1.06 (0.94-1.19) | 0.02 (-0.02-0.06) | - |
| 60-69 years | 0.57 (0.039) | 0.46 (0.037) | 1.29 (1.18-1.42) | 0.13 (0.08-0.19) | 114 (90-138) |
| 70-79 years | 0.67 (0.047) | 0.51 (0.045) | 1.34 (1.21-1.48) | 0.17 (0.11-0.24) | 251 (202-301) |
| 80+ years | 0.69 (0.058) | 0.46 (0.059) | 1.54 (1.34-1.75) | 0.25 (0.16-0.34) | 388 (297-479) |

Note: Relative risks from matched case-control analyses- adjusted for number of hospitalisations, GP visits, prescriptions, and ED visits within a period of 1 year prior to the pre-pre-acute phase; age; sex; residency, partner/married, smoking, attended preventative health screening, taken supplements 4 weeks prior to recruitment, prior immune suppression, prior cancer, physical limitation, heart disease and asthma.

^a^ These analyses include hospitalisation in only those cases of HZ which were diagnosed on the basis of prescription of specific antiviral medications, N=5847.

^b^ These analyses included total claims recorded on MBS

Abbreviations: SE, standard errors; CI, confidence interval; MBS, Medicare Benefits Schedule

Appendix E: Number of prescriptions per 1000 participants (analgesics, antidepressants (including amitriptyline), and anticonvulsants)

Note: The vertical dotted line represents the day of herpes zoster diagnosis

Appendix F: Excess hospitalisations, general practitioner visits, prescriptions filled, and emergency department visits per herpes zoster case during the acute/sub-acute period (between 21 days before and until 90 days after the onset of herpes zoster) calculated in self-matched analyses (see Appendix II for methods).

| Patient group | Hospitalisations (95% CI) | General practitioner visits (95% CI) | Prescriptions filled (95% CI) | Emergency visits (95% CI) |
| --- | --- | --- | --- | --- |
| Age groups (years) |  |  |  |  |
| Overall | 0.07 (0.04-0.11) | 1.57 (1.49-1.67) | 2.18 (1.18-1.72) | 0.11 (0.09-0.14) |
| 45-59 | 0.03 (-0.02-0.10) | 1.34 (1.19-1.51) | 1.16 (0.85-1.50) | 0.07 (0.04-0.12) |
| 60-69 | 0.07 (0.02-0.14) | 1.56 (1.39-1.72) | 2.21 (1.62-2.72) | 0.14 (0.09-0.20) |
| 70-79 | 0.08 (0.01-0.16) | 1.72 (1.54-1.96) | 3.23 (2.45-3.88) | 0.13 (0.08-0.18) |
| ≥80 | 0.16 (0.03-0.34) | 1.86 (1.54-2.12) | 2.86 (1.90-3.97) | 0.17 (0.10-0.27) |

Abbreviations: CI, confidence interval
